# Arbuscular mycorrhizal fungi and salinity stress mitigation in plants

**DOI:** 10.3389/fpls.2024.1504970

**Published:** 2025-01-17

**Authors:** Mohammad Reza Boorboori, Lenka Lackóová

**Affiliations:** ^1^ College of Environment and Surveying and Mapping Engineering, Suzhou University, Suzhou, Anhui, China; ^2^ Faculty of Horticulture and Landscape Engineering, Institute of Landscape Engineering, Slovak University of Agriculture in Nitra, Nitra, Slovakia

**Keywords:** mycorrhiza, salinity, plant, resistance, symbiotic relationship

## Abstract

In recent decades, climate change has caused a decrease in rainfall, increasing sea levels, temperatures rising, and as a result, an expansion in salt marshes across the globe. An increase in water and soil salinity has led to a decline in the cultivated areas in different areas, and consequently, a substantial decrease in crop production. Therefore, it has forced scientists to find cheap, effective and environmentally friendly methods to minimize salinity’s impact on crops. One of the best strategies is to use beneficial soil microbes, including arbuscular mycorrhizal fungi, in order to increase plant tolerance to salt. The findings of this review showed that salinity can severely impact the morphological, physiological, and biochemical structures of plants, lowering their productivity. Although plants have natural capabilities to deal with salinity, these capacities are limited depending on plant type, and variety, as well as salinity levels, and other environmental factors. Furthermore, result of the present review indicates that arbuscular mycorrhizal fungi have a significant effect on increasing plant resistance in saline soils by improving the soil structure, as well as stimulating various plant factors including photosynthesis, antioxidant defense system, secondary metabolites, absorption of water and nutrients.

## 1 Introduction

Many environmental stresses adversely affect plants’ metabolisms and growth, which ultimately impacts their performance (Hashem et al., 2018). The salt stress is one of the most significant abiotic stresses around the globe, and it has caused severe ecological (including the reduction of biological diversity, destruction of pastures and forests, desertification, and soil erosion) and agricultural problems, particularly in arid and semiarid regions (Himabindu et al., 2016; Jia et al., 2019). Global warming and climate change are contributing to soil salinity by reducing rainfall and increasing transpiration and evaporation, which, along with unsustainable farming practices (including chemical overuse, and watering with saline water), have led to the spread of salt marshes worldwide (Guo et al., 2020; Trenberth et al., 2014). This phenomenon, which is expanding by creating a continuous shortage of atmospheric and pedosphere water, has caused a reduction in the quality and quantity of agricultural products, damage to agricultural land, and a decrease in farmland, and strongly affects human food security and diet (Chaves et al., 2009; Estrada et al., 2013; Ashraf and Harris, 2013; Pan et al., 2020).

According to their salt sensitivity, plants are classified in two major groups: halophytes and glycophytes (Yuan et al., 2019; Pan et al., 2020). Plants called halophytes are salt-tolerant plants that can thrive in soil or water with high saline concentrations (200 mM NaCl or more), whereas glycophytes are salt-sensitive (Wang et al., 2019a; Himabindu et al., 2016). Glycophytes are the predominant crop and forage species used in modern agriculture, which have limited mechanisms of salt tolerance, whereas halophytes have effective mechanisms for protecting themselves from salt damage (Flowers and Muscolo, 2015; Pan et al., 2020). Nevertheless, at the beginning of the growth process, both plants are sensitive to salinity (Himabindu et al., 2016).

Food security for the world’s expanding populace in the face of deteriorating agricultural land is one of humanity’s most important missions, which requires effective strategies to mitigate salinity (Chinnusamy et al., 2005; Duc et al., 2021), this includes the cultivation of salt-resistant crops, adding solutes and growth regulators, implementation of better irrigation systems, plant breeding, and supplying plant growth promoting microorganisms (PGPM) (Khan et al., 2010; Munns, 2002; Diagne et al., 2020). Among the methods mentioned above, the inoculation of plants by beneficial soil microorganisms is an efficient method that, in addition to increasing plant resistance against salt stress, also improves their productivity (Janah et al., 2021b; Ait-El-Mokhtar et al., 2019).

Among the PGPM, arbuscular mycorrhizal fungi are crucial because they establish symbiosis with 80% of plants, including glycophytes, and halophytes (Khan et al., 2010; Gao et al., 2020). Arbuscular mycorrhizal fungi survive in most environments, and provide a variety of ecological services, especially enhancing the rhizosphere properties chemically and physically, strengthening the ecosystem function, and increasing host plant growth and performance (Mathur et al., 2019; Liang et al., 2021; Frosi et al., 2018). Coexistence with mycorrhizal fungi, which is known as the “mother of all plant root symbioses,” is one of the most common strategies used to resist abiotic, and biotic stresses (Romero-Munar et al., 2017). Since mycorrhizal fungi are discovered throughout the planet, including in highly salty conditions, their association with plant roots can be a valuable ecological method to sustain plants in salinity environments (Aroca et al., 2013; Evelin et al., 2009; López-Ráez, 2016).

The mycorrhizal fungi are capable of regulating numerous physiological and biochemical processes within plants, and reduce salinity’s negative effects on them (Augé et al., 2014; Ait-El-Mokhtar et al., 2019). Among the processes that arbuscular mycorrhizal fungi regulate in host plants, and enhance their flexibility in salinity-stressed conditions are the following: facilitating water and nutrient absorption, enhancing photosynthetic ability, modulating antioxidant responses, inhibiting ion absorption, increasing root and shoot biomass, expression of aquaporin genes, gene expression encoding membrane transport proteins, and accumulating compatible solutes (Hashem et al., 2018; Chen et al., 2017; Chang et al., 2018; Ding et al., 2016; Chaichi et al., 2017). Plants that are salt-tolerant under mycorrhizal fungi treatment include soybean, sorghum, wheat, tomato, rice, watermelon, cucumber, safflower, and pistachio (Abbaspour et al., 2021; Diao et al., 2021). The purpose of this review is to describe, existing knowledge about the interaction of crops with arbuscular mycorrhizal fungi and salt stress resistance is explored, to make the findings and unknowns regarding the current topic available to researchers for future studies. In this review, the effect of salt stress on the morphophysiological and biochemical structures of plants has been studied. Furthermore, it has been attempted to investigate the recent findings in the field of the Plants’ natural ability to deal with salinity. This review also discusses the mycorrhizal fungi role in increasing plant morpho-physiological, and biochemical structure, as well as its role in enhancing plant performance in saline environments.

## 2 Salinity

Plants in nature are confronted with various stresses, and one of the main abiotic stresses is salinity that limit the growing, development, and metabolism of plants (Abdel Latef and Chaoxing, 2014; Diagne et al., 2020). Land degradation due to soil salinization has become a deteriorating environmental crisis in farming ecosystems, especially in arid and semiarid areas, and has severely impacted food security worldwide (Porcel et al., 2012; Chen et al., 2017; Van Zelm et al., 2020; Deinlein et al., 2014). It is estimated that one billion hectares worldwide are influenced by saline conditions, and approximately, salinization is affected 10% of arable land (Tufail et al., 2018; Duc et al., 2021; Zaman et al., 2018). Areas affected by salinity are increasing by 15 to 20 million hectares per year, which reduces production of crops by 20% (Evelin et al., 2019; Pan et al., 2020), and by 2050, it is expected that half of arable land could be negatively impacted by salinization due to climate change (Porcel et al., 2012; Janah et al., 2021b; Diao et al., 2021). Soil salinity has impacted agricultural productivity in a number of countries, including Iran, Pakistan, Thailand, Iraq, China, Egypt, India, Australia, Argentina, and the United States more than in other places around the world (Rengasamy, 2010; Abeer et al., 2014; Shrestha, 2006). There are roughly 100 million hectares of salt marshes in China (Huang, 2018), while 1.84 million hectares of saline soil exist in the northeastern regions of Thailand (Arunin and Pongwichian, 2015). 55% of Senegal’s arable land and nearly 37% of Morocco’s soil are impacted by salt (Diagne et al., 2020; Farissi et al., 2011).

Salinity in soil can be caused by different kinds of salts, such as NaCl, Na_2_CO_3_, MgCl_2_, CaSO_4_, MgSO_4_, and Na_2_SO_4_, among which sodium chloride is most common in arid and semiarid soils (Flowers et al., 1977). Saline soils have an electrical conductivity (EC) of more than 4 decisiemens per meter (dS/m), and an osmotic pressure of -0.2 MPa, which is equal to about 40 mM NaCl (Santander et al., 2019). Due to salinity, the soil’s biological, chemical, and physical properties are destroyed, decreasing fertility and increases desertification (Diagne et al., 2020; Romero-Munar et al., 2019). A high salt concentration in soil solution reduces the osmotic potential of the soil and also suppresses the activities related to nutrients in plants, and leads to unfavourable Na^+^/Ca^2+^ and Na^+^/K^+^ ratios (Romero-Munar et al., 2019; Kumar and Verma, 2018) (**Table 1**).

**Table 1:** Soil classification based on pH, EC, SAR, and ESP.

Classification	pH of the soil	Electrical Conductivity (EC) (dS/m)	Sodium Adsorption Ratio (SAR)	Exchangeable Sodium Percentage (ESP)
Saline-Sodic	< 8.5	> 4.0	> 13	>15
Sodic	> 8.5	< 4.0	> 13	>15
Saline	< 8.5	> 4.0	< 13	< 15
No Salt Affects	< 8.5	< 4.0	< 13	< 15

Nomenclatureis as proposed by Pastia et al. (2016).

Soil salinity is the result of natural factors such as capillary rise due to evaporation, seawater infiltration, dissolution of rocks, and lack of rainfall, as well as human factors like poor agricultural practices, including improper irrigation and drainage systems (Legros, 2009; Latef and Chaoxing, 2011; Wang et al., 2003). A rise in soil salt levels causes osmotic, and ionic stress in plants, directly reducing crop productivity by disrupting important biochemical, and physiological processes (Abd_Allah et al., 2015). Therefore, in addition to agricultural production, salinity also threatens ecosystem performance (Santos et al., 2016). Thus, developing effective methods for the restoration of saline lands despite the climatic and economic limitations seems to be necessary, Including 1) biological control through the restoration of saline soils with organic materials and cultivation of halophytes, 2) chemical control by applying chemical amendments such as sulfuric acid, sulfur, and gypsum to neutralize alkaline condition, and 3) mechanical control by installation of anti-salt structures such as dams (Fall et al., 2018; Litalien and Zeeb, 2020).

## 3 Salinity in plants

**Figure 1:**
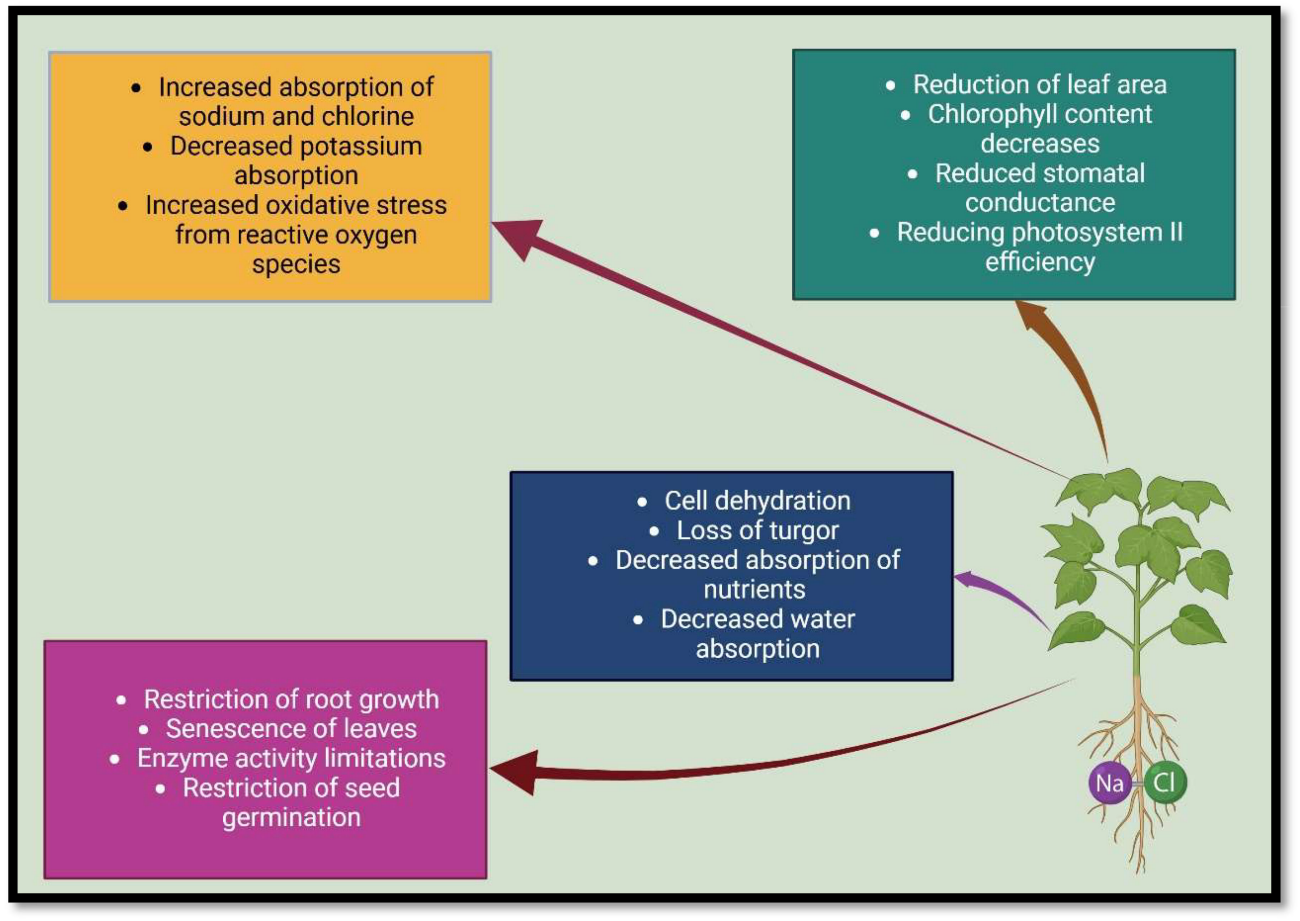
Salinity stress changes plant morphology and physiology.

Too much salt in the soil disrupts all the morphological, physiological, and biochemical reactions in plants and drastically reduces crop production (Nawaz et al., 2010; Aroca et al., 2013; Balliu et al., 2015). Salinity hurts plants through three physiological aspects (Zheng et al., 2008; Boorboori, 2023), which are: 1) osmotic tension: increasing the salt concentration in the soil changes the basic texture, and reduce water conductivity, aeration of the soil, and a decrease in osmotic potential, leading to a physiological dryness in plants, as well as the nutritional imbalance (Evelin et al., 2012; Hashem et al., 2016; Chaichi et al., 2017), 2) toxic ions: the overabsorption of toxic ions, including sodium (Na^+^), and chlorine (Cl^−^) by plants can result in changes to enzyme structure, damage to cell organelles, decrease the activity of metabolic enzymes, change in macromolecule structure, inhibition of photosynthesis, protein synthesis inhibition, and ion balance disruption (Porcel et al., 2012; Boorboori and Zhang, 2023), and 3) oxidative damage: an increase in soil salinity causes secondary stress in plants called oxidative stress, which leads to disturbances in plant cell structures such as mitochondria, chloroplasts, membranes, etc. (Abdel Latef et al., 2019; Kumar et al., 2015; Porcel et al., 2012) (**Figure 1**).

### 3.1 Absorption of water and nutrients by plants

**Figure 2:**
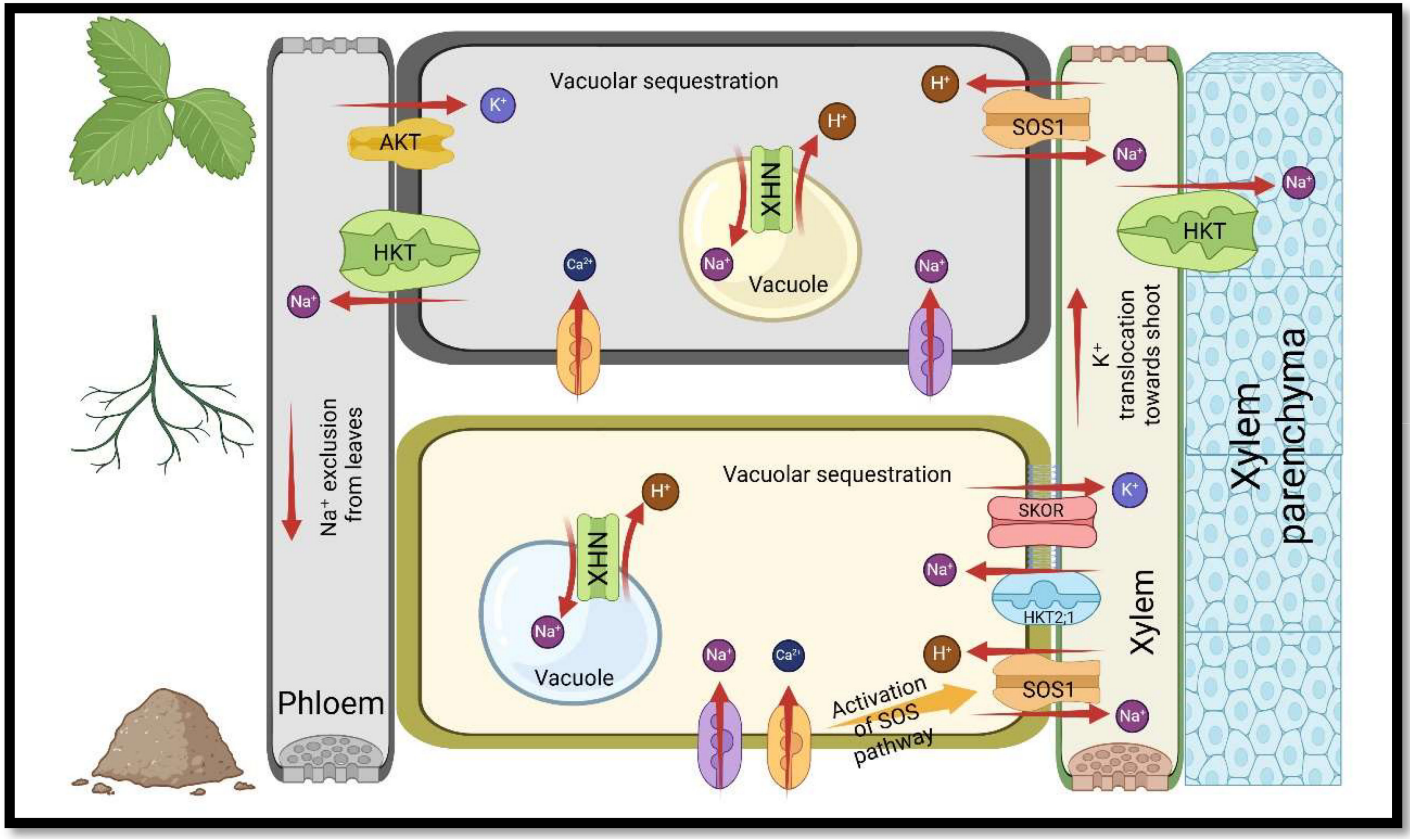
The function of transporter proteins in sustaining acceptable K^+^/Na^+^ ratios in plants under salt stress. Nomenclature is as proposed by Evelin et al. (2019), and Boorboori and Zhang (2022).

Since salts are also plant nutrients, excess salt in the soil imposes competition during the absorption, transfer, or distribution of nutrients, leading to an unbalance in the plant ionic composition, and thus affecting the physiological characteristics of the plant (Rabie and Almadini, 2005; Farissi et al., 2011; Munns and Tester, 2008). Studies in saline environments have indicated that an abundance of Na^+^ and Cl^−^ ions hinder the solubility, mobility and absorption of nutrients, for instance, nitrogen (N), phosphorus (P), potassium (K), calcium (Ca), magnesium (Mg), iron (Fe), copper (Cu), and zinc (Zn) in plants (Zai et al., 2021; Hasegawa et al., 2000; Evelin et al., 2019). Na^+^ can enter the roots through non-selective cation channels (NSCCs) in the plasma membrane, increasing Na^+^ content in roots and shoots, and leading to unfavourable Ca^2+^/Na^+^ and K^+^/Na^+^ ratios (Abdel-Fattah and Asrar, 2012; Van Zelm et al., 2020; Pedranzani et al., 2016). Since Na^+^ competes with K^+^ and Ca^2+^ for membrane transfer sites, maintaining a high ratio of Ca^2+^/Na^+^ and K^+^/Na^+^ in the cytosol is crucial for increasing plants’ tolerance to salinity and enhancing enzymatic processes (Evelin et al., 2019; Ahanger et al., 2015; Samaddar et al., 2019). A reduction in the ratio of K^+^/Na^+^ in the cytosol causes a disturbance in stomatal movement, turgor maintenance, photosynthesis, activity of enzymes, and protein synthesis (Benito et al., 2014; Maathuis and Amtmann, 1999). In contrast, a decrease in the ratio of Ca^2+^/Na^+^ causes photosynthetic tissue destruction, disruption of Ca^2+^ signalling pathways, and a reduction in hydraulic conductivity (Ait-El-Mokhtar et al., 2020) (**Figures 1**, **2**).

N is an essential macronutrient that plants absorb it as nitrate (NO_3_-), and ammonium (NH_4_^+^) ions, whereas salinity interferes with their absorption by immobilizing them (Frechilla et al., 2001; Munns and Tester, 2008). In the saline environment, the NH_4_^+^ uptake is challenged by Na^+^, whereas the NO_3_- uptake is inhibited by Cl^−^ in the membrane, which causes the low flux of NH_4_^+^ and NO_3_- from soil to roots, leading to a reduction in nitrate reductase activity (NR) in plants (Hoff et al., 1992; Evelin et al., 2019). P is another nutrient whose absorption by roots is interfered with salinity because of its precipitation with other cations, for example, Zn2^+^, Mg2^+^, and Ca2^+^ (Bano and Fatima, 2009; Evelin et al., 2019). The decrease in P content as an essential macronutrient in plants causes the stoppage of growth and premature death of leaves (Giri et al., 2003; Taiz et al., 2015). Furthermore, salt in the soil renders water unavailable to plant roots, resulting in a decline in turgor pressure in plant cells, cell dehydration, and water stress (Füzy et al., 2008; Zou et al., 2013; Ait-El-Mokhtar et al., 2020) (**Figure 2**).

A plant’s root system absorbs water and nutrients, and it also comes in direct contact with a salty environment, so it contributes greatly in protecting the plant from salinity stress (Rana et al., 2019; Evelin et al., 2019). There is a decrease in growth of roots with increasing salinity of soil, and this is the first response of plants to excessive soil salts (Amanifar et al., 2019; Greenway and Munns, 1980). When salt is present in the rhizosphere, primary roots grow slower because salt inhibits cell division and root epidermal cells elongation, while lateral roots grow (Bernstein et al., 2013; Jung and McCouch, 2013). Furthermore, there is evidence that the toxic effects of salinity on root growth and development are related to the inhibition of endogenous phytohormones levels, such as Indole-3-acetic acid (IAA), and indole-3-butyric acid (IBA) (Khan et al., 2015; Egamberdieva, 2009; Egamberdieva et al., 2016).

### 3.2 Photosynthesis

Photosynthesis, as a primary metabolism’s key process, is highly sensitive to salinity (Jia et al., 2019; Porcel et al., 2012), and salinity of the soil inhibits plant photosynthetic ability through its effect on leaf surface area, photosystem II efficiency, stomatal conductance, and content of chlorophyll (Farissi et al., 2011; Miransari et al., 2008). Salinity directly damages the complete ultrastructure of photosynthetic organelles such as chloroplasts and also reduces photosynthetic pigment content (total chlorophyll, carotenoids, Chl a and Chl b) (Liang et al., 2021; Akram and Ashraf, 2011; Ait-El-Mokhtar et al., 2020). Chloroplasts are the most susceptible cellular organelles to salt, and in high salinity conditions, grana and thylakoids begin to disintegrate, and vanish as a result of concentration of cations changes in chloroplasts, membrane damage and swelling of thylakoids, which consequently affects the efficiency of light energy utilization (Kaya et al., 2009; Peng et al., 2019). According to studies, salinity suppressed enzymes involved in the synthesis of photosynthetic pigments, including chlorophyll synthetase, and increased chlorophyll degradation enzyme activity, reducing chlorophyll and content of photosynthetic pigments (Wang et al., 2019a; Zai et al., 2021; Tsunekawa et al., 2009). In addition, under salinity stress, chlorophyll levels can decrease due to low mineral absorption, particularly magnesium (Giri and Mukerji, 2004). However, photosynthetic pigment synthesis, and efficiency of photosynthesis in saline environments strongly depends on the plant species and even the genotypes (Bistgani et al., 2019; Khan et al., 2015) (**Figure 1**).

In addition to directly damaging photosynthetic machinery, salt stress can also damage the photosystem II (PSII) reaction center, interfere with electron transfer from PSII to photosystem I (PSI), and ultimately decrease photosynthesis (Evelin et al., 2013; Liu and Shi, 2010; Jia et al., 2019). Furthermore, the researchers found that under salinity stress potential photochemical efficiency (Fv/Fo), maximum quantum efficiency of PSII (Fv/Fm), and photochemical quenching coefficient (qP) decreased while non-photochemical quenching (NPQ) increased (Liang et al., 2021; Sheng et al., 2008). Osmotic stress caused by salinity reduces the water content in shoots and leaves, thus closing the stomata and reducing CO_2_ availability (Gao et al., 2020; Duarte et al., 2013; Zu et al., 2005). In the absence of CO_2_ absorption, excess electrons are accumulated in the thylakoid membranes (disruption of electron transfer between PSII and PSI), resulting in PSII degradation and damage to other photosynthetic apparatus components (Gao et al., 2020; Chang et al., 2018).

### 3.3 Reactive oxygen species

**Figure 3:**
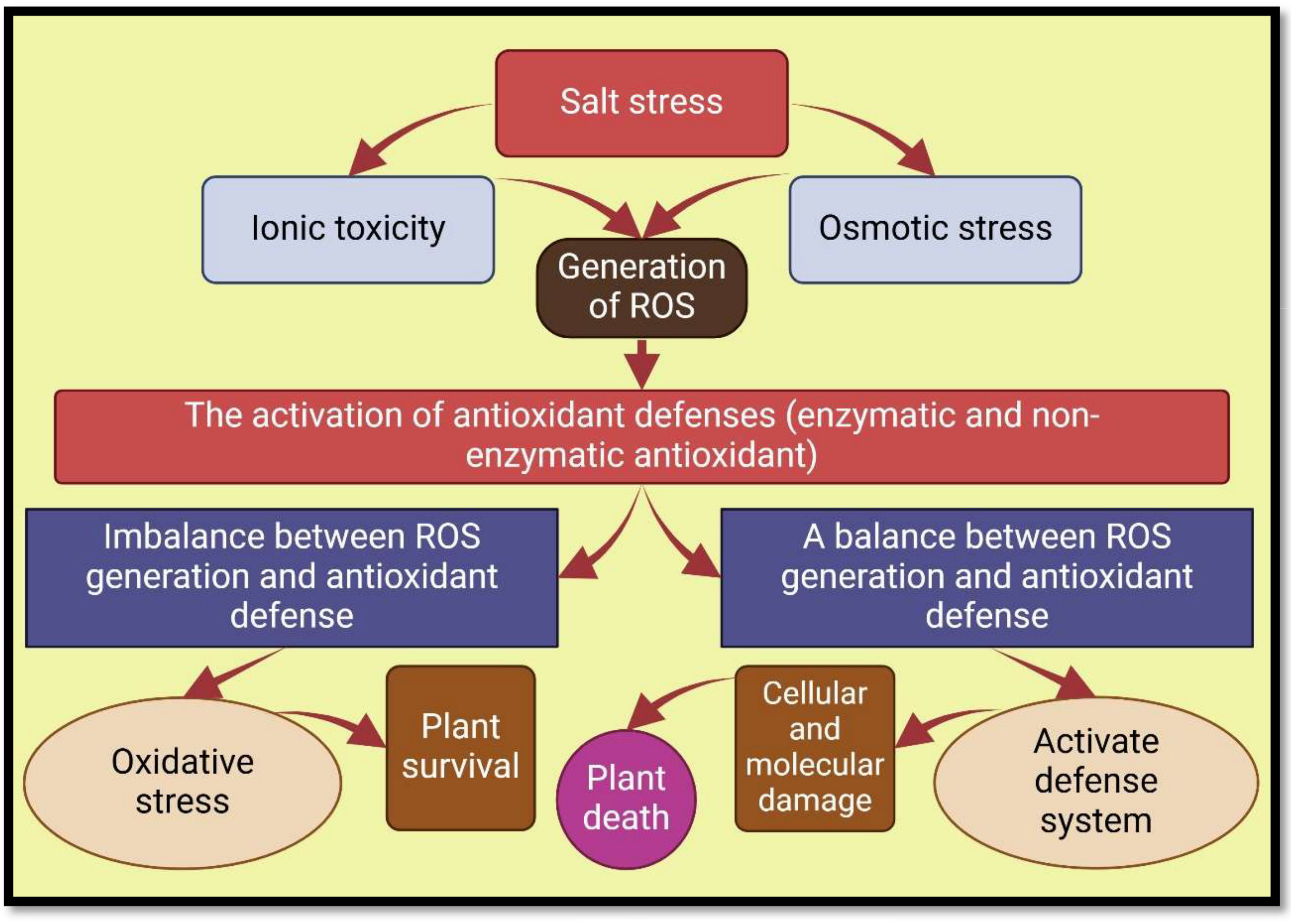
Oxidative stress, and antioxidant defenses in salt-exposed plants. Nomenclature is as proposed by Hasanuzzaman et al. (2021).

Salt-induced stress leads plants to produce an excessive amount of reactive oxygen species (ROS), which causes oxidative degradation to components of cells (Amanifar et al., 2019; Parihar et al., 2020; Boorboori and Zhang, 2022). Oxidative stress process in plants occurs when multiple metabolic pathways are disrupted, and high-speed and energy electron transfers to molecular oxygen (Gill and Tuteja, 2010; Miller et al., 2010). Oxidative damage caused by ROS to plants includes hydroxyl radicals (·OH), superoxide radicals (O_2_^•−^), hydrogen peroxide (H_2_O_2_), and singlet oxygen (1O_2_) (Abd_Allah et al., 2015; Gao et al., 2020). ROS buildup disrupts important cellular structures, including organelles, membrane lipids, nucleic acids, proteins, macromolecules (especially enzymes), leading to a reduction in nutrient absorption and transfer, disruption of respiratory and photosynthesis systems, and ultimately limiting growth and development of plants (Porcel et al., 2016; Janah et al., 2021b; Abeer et al., 2014) (**Figures 1**, **3**).

Lipid peroxidation changes cell membrane’s selective permeability, and eventually causes membrane leakage, and membrane integrity loss (Abeer et al., 2014; Huang et al., 2010). Maintaining the integrity of the cell membrane is essential for plants to cope with salinity stress, since the damaged cell membrane loses its biological function and affects plant’s natural metabolism (Stevens et al., 2006; Jabeen et al., 2014). According to Nat et al., cell membranes of halophytes can be damaged by peroxidation of lipids when salt concentrations are above 300 mM for a week or more (Nath et al., 2016). Malondialdehyde content (MDA) as lipid peroxidation’s final product, in combination with relative electrolyte leakage (REL) is used as an oxidative damage index, and to identify cell membrane damage extent (Adolfsson et al., 2017; Pedranzani et al., 2016).

### 3.4 Plants’ adaptation to salt stress

Plants contain a very flexible system for adjusting their morpho-physiological, molecular, biochemical and metabolic mechanisms for survival in salinity environments (Porcel et al., 2012; Evelin et al., 2019). Plants enhance their resistance against stress salinity by raising the accumulation of compatible osmolytes, regulating water absorption, maintaining the endogenous levels of growth regulators, increasing chlorophyll synthesis, regulating antioxidant molecules, and compartmentalizing toxic ions in vacuoles (Bistgani et al., 2019; Abbaspour et al., 2021; Munns and Tester, 2008). Additionally, plants regulate the rate of leaf transpiration, stomatal conductance, and photosynthesis rate by adjusting the stomata aperture, leading to greater photosynthesis efficiency (Kaya et al., 2009; Elhindi et al., 2017; Liang et al., 2021).

Osmotic regulation is another defense mechanism that helps plants control osmotic and ionic toxic impacts through the expression of genes involved in nutrient transport and partitioning, accumulation of solutes, and aquaporins (Li et al., 2020; Apse and Blumwald, 2007; Van Zelm et al., 2020). Salt Overly Sensitive 1 (*SOS1*) is a Na^+^/H^+^ antiporter in plasma membrane, which has an essential function in maintaining ions homeostasis and managing Na^+^ and K^+^ transport in plasma membrane and tonoplast (Li et al., 2020; Gao et al., 2020). The study conducted on *Suaeda salsa* showed that at 400mM NaCl concentration, Na^+^ was actively removed from the cytoplasm into the rhizosphere, while the expression of *SOS1* was the highest in the roots (Gao et al., 2020). Furthermore, vacuolar Na^+^/H^+^ antiporters (*NHX*s) are responsible for the sequestration of Na^+^ into vacuoles, which is driven through the proton motive force generated by H^+^-PPase and H^+^-ATPase (VHA) (Diao et al., 2021; Gao et al., 2020). Moreover, The Stelar K^+^ outward rectifier channel (*SKOR*) is a K^+^ channel in plants that transports K^+^ from the roots to aerial parts (Li et al., 2023). *SKOR* identify as a transport protein with a role in loading K^+^ to the xylem, and if it was disrupted K^+^ content in shoots was significantly reduced, while K^+^ content in roots was not affected (Abbaspour et al., 2021; Sharma et al., 2013). Moreover, plasma membrane intrinsic proteins (*PIPs*), a plant’s aquaporin subfamily, was found to be the primary water absorption and transport channels in plant cells and can mediate the plant cell water loss under the stress of salt (Abbaspour et al., 2021).

Increasing the production of ROS can be harmful to cells, but they also regulate numerous fundamental plant processes, including salt stress response (Chen et al., 2020). Nevertheless, plants have evolved detoxification mechanisms to combat oxidative damage, including the induction of secondary metabolite production and a wide range of enzymatic and non-enzymatic antioxidants (Behdad et al., 2020; Toscano et al., 2019; Sharma et al., 2012). The antioxidant enzymes that reduce salinity-induced ROS include catalase (CAT), peroxidase (POD), superoxide dismutase (SOD), ascorbate peroxidase (APX), glutathione reductase (GR), and polyphenol oxidases (PPO) (Janah et al., 2021a; Waszczak et al., 2018; Apel and Hirt, 2004). In contrast, non-enzymatic antioxidants that destroy ROS include glutathione (GSH), ascorbic acid (AsA), carotenoids, flavonoids, and α-tocopherol (Foyer and Noctor, 2011; Chaparzadeh et al., 2004).

Salt stress stimulates phenolic acid, phenolic compounds, and flavonoids accumulation in plants as stress-resistance mechanism (Boughalleb et al., 2020; Duc et al., 2021; Liang et al., 2021). A change in the accumulation of phenolic compounds may be related to changes in other metabolic processes, such as phenolic acids, fatty acids, organic acids, amino acids (Saleh et al., 2020), and sugars, which leads to an improvement in the defense system of the plant, ROS reduction, and osmotic regulation (Abeer et al., 2014; Arzani and Ashraf, 2016; Saxena et al., 2015). Further, the increase in total phenolic acids is mainly due to the slope of quinic, protocatechuic, and gallic acids, followed by epicatechin, catechin, and quercetin-3-O-galactoside (Boughalleb et al., 2020). Additionally, plants are able tolerating salt by osmolytes accumulation in their cell cytoplasm for osmotic regulation, including polyamines (spermine, spermidine, and putrescine), proline, glycine, organic acids, total suspended solids (maltose, dextrin, sucrose, and glucose), α-amino nitrogen, and betaine (Evelin et al., 2013, 2019; Dodd and Pérez-Alfocea, 2012; Chen and Murata, 2011; Yang and Guo, 2018).

## 4 Arbuscular mycorrhizal fungi and salinity stress in plants

**Figure 4:**
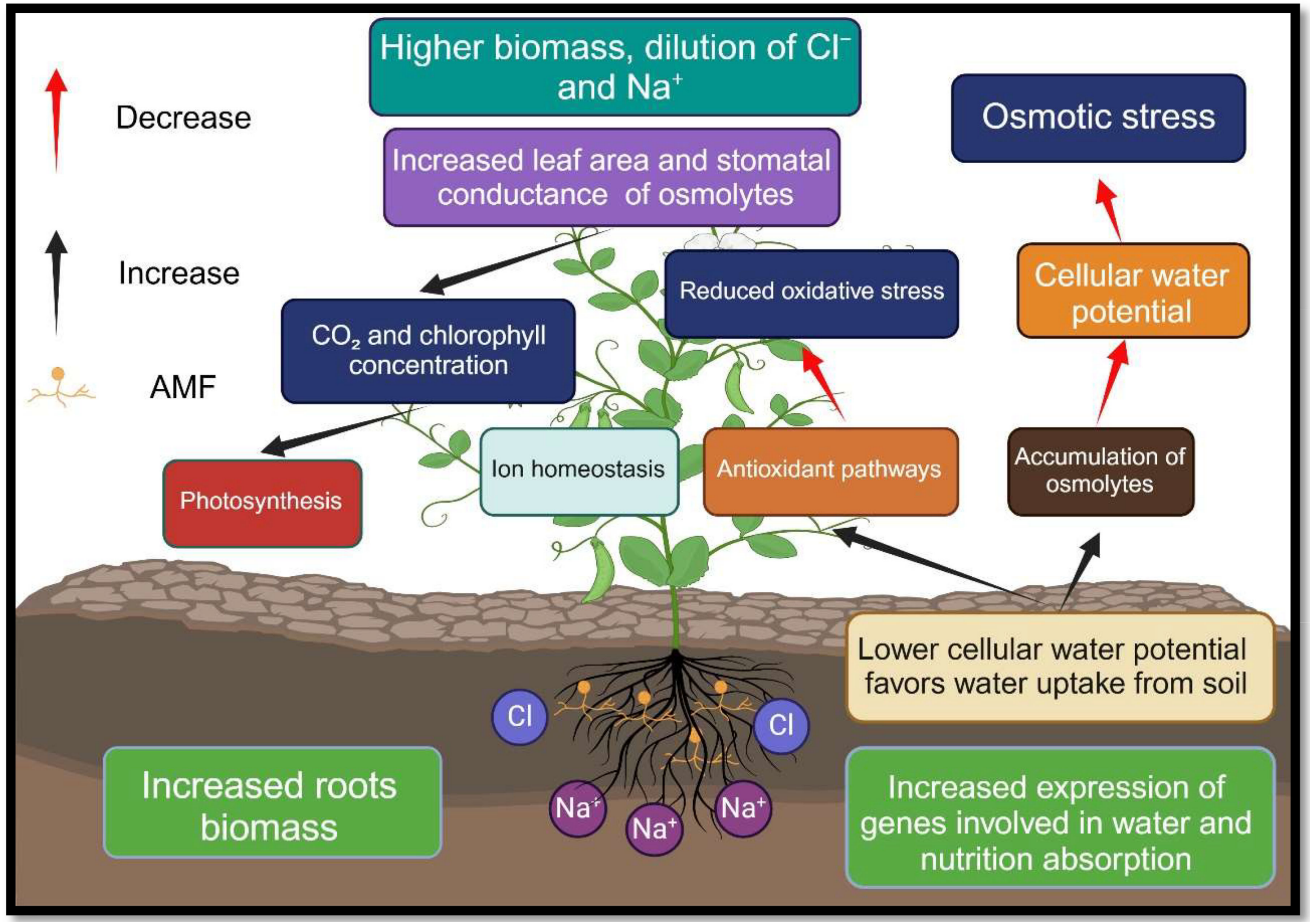
Advantages of colonizing plant roots with arbuscular mycorrhizal fungi in saline soils. Nomenclature is as proposed by Gupta et al. (2021).

The AMF are a common soil microbe that colonizes the roots of most terrestrial plants (Duc et al., 2021). According to studies, these symbiotic fungi provide significant benefits to their host plants, including growing their resistance to stress, especially salt stress (Duc et al., 2021; Evelin et al., 2019). AMF increases plant resistance to salt by various biochemical and physiological mechanisms, which can be categorized into three groups: 1) increasing nutrient absorption and maintaining ionic homeostasis in plants, and improving water absorption and maintaining osmotic balance, 2) enhancing photosynthesis efficiency and protecting the photosynthetic apparatus, and 3) plant hormone profile modulation and antioxidant system induction to prevent ROS damage (Evelin et al., 2019; Tavarini et al., 2018; Smith et al., 2010) (**Figure 4**).

AMF can adapt and survive in a salty habitat, however, high salt stress may inhibit spore germination, reduce spore viability, prevent hyphae growth, decrease spore density, and generally diminish AMF biomass (Ahanger et al., 2014; Juniper and Abbott, 2006; Hammer et al., 2011; Estrada et al., 2013). Moreover, Na^+^ exerts a direct toxic affects AMF, and reduces colonization rate, indicating suppression of the AMF symbiotic effect by salinity (Sheng et al., 2008; Chaichi et al., 2017). According to research on licorice, it was discovered that salinity greatly reduced the ability of *Funneliformis mosseae* to infect the roots (Amanifar et al., 2019). High level of salt reduces AMF colonization percentage by reducing mycelium growth, vesicles and arbuscules in plants (Bistgani et al., 2019; Abeer et al., 2014; Rivero et al., 2018). Nevertheless, the adverse effect of excessive salt exposure on capacity of AMF colonization is greater in plant growth in its early stages, however, AMF eventually adapts to such a salt level over time (Duc et al., 2021). A study conducted on *Eclipta prostrata* L. was shown that in high-salinity environments (200 mM NaCl), the colonization of different AMF species (*Acaulospora lacunose*, *Septoglomus deserticola*, and *Funneliformis mosseae*) was significantly less in the first four weeks of growing a plant, however, colonization rates increased in the later stages of plant growth (Duc et al., 2021).

There are a number of factors that determine whether AMF symbiosis with plants increases in saline soil, including the type of plant, AMF genotype, and the external agro-environment (Johnson et al., 2015; Estrada et al., 2013). In general, AMF inoculation efficiency increases among plant species with salinity tolerance capabilities (Fan et al., 2012; Aliasgharzadeh et al., 2001). Additionally, monocotyledonous plant species with fibrous root systems can better coexist with AMF than dicotyledonous species with tap root systems (Abd Rahim et al., 2016; Jia et al., 2019). The researchers also found that AMF symbiosis effect is diverse in different organs of a plant [above organs (e.g., leaves) and underground organs (e.g., roots)] exposed to salt (Gao et al., 2020). Meanwhile, native AMF ecotypes isolated from saline environments maintain higher colonization characteristics compare to the salt-sensitive genotypes, and show a greater level of salinity resistance in plants (Bistgani et al., 2019; Samaddar et al., 2019; Campagnac and Khasa, 2014; Yamato et al., 2008).

Several studies have shown that combining AMF with other beneficial soil microorganisms increases plant resistance to salinity, however, species closely related to AMFs with similar phenotypic characteristics compete fiercely for limited space and resources with them (Ait-El-Mokhtar et al., 2019; Maherali and Klironomos, 2007). Additionally, according to previous research, some compounds affect AMF symbiosis, including dopamine, hydroxyl fatty acids, phenols, sesquiterpenoids, and flavonoids (Gao et al., 2020; Lanfranco et al., 2018). Numerous reports indicate AMF inoculation reduces salt stress in different plants, including lettuce, alfalfa, tomato, wheat, maize, oleaster, black locust, rice, castor, peanut, swamp she-oak, mandarin, basil, cucumber, lychee, *Panicum turgidum*, *Senegalia senegal*, *Acacia mangium*, *Acacia auriculiformis*, and etc (Diagne et al., 2020; Hashem et al., 2018; Guo et al., 2020; Tisarum et al., 2020; Jia et al., 2019; Santander et al., 2019; Pan et al., 2020; Elboutahiri et al., 2010).

### 4.1 AMF improves the uptake of nutrients and water in salt-stressed plants

The mutual interaction between AMF, and salt-stressed plants enhances the selective absorption of some elements (Ca, K), limits the absorption of some other elements (Na^+^), increases the efficiency of water consumption, and promotes host plant growth (Hammer et al., 2011; de Varennes and Goss, 2007; Santander et al., 2017). In saline environments, plant root and soil colonization by AMF can improve the rhizosphere condition of the soil through strengthening the absorption of organic carbon in the soil, increasing N, P and K pools, improving the content of organic matter, adjusting pH of the soil, and preventing soil erosion (Zai et al., 2021; Chang et al., 2014; El Kinany et al., 2019; Aliasgharzadeh et al., 2001). Moreover, AMF improves water and nutrient absorption for host plants by forming a wide hyphal network and spreading myciniums outside the rhizosphere (Ortiz et al., 2015; Lin et al., 2016; Yan et al., 2016) (**Figure 4**; **Table 2**).

**Table 2:** The effect of different arbuscular microbial varieties on the decrease of salinity impact in different plants.

	Plant	Arbuscular mycorrhiza variety	Salinity unit	The effect of Arbuscular mycorrhiza on plants stressed by salt	Reference
1	*Lactuca sativa* var. longifolia	*Claroideoglomus claroideum*	0. 40 and 80 mM	Increase in biomass	(Santander et al., 2019)
				Increased proline synthesis	
				Increase absorption of nutrients	
				Improve ionic balance	
				Keeping roots free of toxic sodium ions	
**2**	*Glycine max* L. Merrill	*Funneliformis mosseae* (syn. *Glomus mosseae*),	100, 200, and 300 mM	Enhancing the formation of nodules	(Bistgani et al., 2019)
				Improve the content of leghemoglobin	
				Enhance the activity of nitrogenase	
		*Rhizophagus intraradices* (syn. *Glomus intraradices*)		Increasing auxin synthesis	
				Protection against membrane damage caused by salt	
	with genotypes Clark (salt tolerant) and Kint (salt sensitive)			Reducing hydrogen peroxide production	
		and *Claroideoglomus etunicatum* (syn. *Glomus etunicatum*)		A reduction in the production of Thiobarbituric Acid Reactive Substances (TBARS)	
				A reduction in the peroxidation of lipids	
				Improve root system	
				Enhance nutrient absorption	
**3**	*Suaeda salsa*	*Funneliformis mosseae*	0, 100, 200 and 400 mM	Increased growth	(Diao et al., 2021)
				Increase in calcium and magnesium concentrations in aerial organs	
				Increase in potassium concentration	
				Reduction of sodium fraction in leaf vacuoles	
				Decreasedthe the expression of *SsNHX1* in shoots and *SsSOS1* in roots at 400 mM NaCl	
				Limit sodium transport from roots to branches	
				It up-regulated the expression of *SsSOS1* in the shoot and down-regulated the expression of *SsSOS1* and *SsNHX1* in the root at 100 mM NaCl	
**4**	*Glycyrrhiza glabra*	*Funneliforms mosseae*	4, 8, 12 and 16 dSm−1	Increasing the concentration of phosphorus and potassium	(Amanifar et al., 2019)
				Increase in shoot proline accumulation	
				Increase in K^+^/Na^+^ ratio	
				Increasing glycyrrhizin concentration	
				Increased beta-amyrin synthase (*bAS*), squalene synthase 1 (*SQS1*), and *P450* genes expression	
				Improve membrane integrity	
				Reduction of ROS production	
				Increase the quality of *Glycyrrhiza glabra* for medicinal purposes	
**5**	*Robinia pseudoacacia* L.	*Rhizophagus irregularis*	0, 100, and 200 mM	Reduction of REL, MDA, and H_2_O_2_ levels in leaves	(Chen et al., 2020)
				Five genes encoding antioxidant enzymes are expressed (*RpGR*, *RpAPX2*, *RpAPX1*, *RpMn-SOD*, and *RpCu/Zn-SOD*) were increased	
				Total, reduced, and oxidized ascorbate and glutathione concentrations increased	
				Increasing the accumulation of H_2_O_2_ and reducing root antioxidant enzyme activities	
**6**	*Arundo donax* L.	*Funnelliformis mosseae*	1, 75, and 150 mM	Increased growth	(Romero-Munar et al., 2019)
				Decreased absorption of NaCl	
				Decreased root-to-stem NaCl transport	
		*Rhizophagus intraradices*		Increasing the efficiency of phosphorus and potassium consumption	
				Decreased Na^+^/K^+^ ratio	
**7**	Oryza sativa L. ssp. *indica* cv. Leum Pua	*Glomus etunicatum*	0 and 150 mM	Increase in dry weight	(Tisarum et al., 2020)
				Decrease in Na^+^/K^+^ratio	
				Flag leaf tissues contain more sucrose	
				Proline and fructose content increased	
		*Glomus geosporum*		Maintaining stomatal function	
				Maintain structure, and function of PSII, Fv/Fm, chlorophyll pigments	
				Cluster numbers, lengths, and weights increased	
				Increase the height of the stem	
		*Glomus mosseae*		Increase in flag leaf length	
				Increased 1000 grains weight	
				Regulation of peonidin-3-glucoside (P3G), and cyanidin-3-glucoside (C3G) in pericarp	
**8**	*Eclipta prostrata* L.	*Funneliformis mosseae*	100 and 200 mM	Peroxidase, and catalase activity increased	(Duc et al., 2021)
		*Septoglomus deserticola*		Increased total phenolic content, and proline	
		*Acaulospora lacunosa*		Increase in 4,5-dicaffeoylquinic acid	
				Increased wedelolactone	
**9**	*Echinacea angustifolia*	*Rhizophagus irregularis*	300 mmol/L	Inhibition of reactive oxygen species	(Jia et al., 2019)
				Increased metabolism of phenylpropane	
				Promotion of protein biosynthesis	
				Acceleration of protein folding	
				Prevent protein degradation	
				Increased ATP synthesis	
				Speeding up photosynthetic electron transport	
**10**	*Malus domestica* Borkh.	*Funneliformis mosseae*	200 mM	Increased carbohydrate content	(Gao et al., 2020)
				Maintaining cell membrane stability	
				Improve photosynthesis	
				Root length, average diameter, forks number, and surface area increased	
**11**	*Echinacea angustifolia*	*Rhizophagus irregularis*	0 and 300 mM	Increase in net photosynthesis	(Liang et al., 2021)
				Increasing plastoglobule number	
				Increasing total flavonoids content	
				Increased leaf proline	
				Reducing the content of malondialdehyde (MDA)	
				Improved PSII performance	
				Improve antioxidant capacity	
**12**	*Stevia rebaudiana* Bertoni	*Rhizophagus irregularis*	80 mM	Increasing antioxidant enzyme activity	(Janah et al., 2021b)
				Improve growth	
				Improve the relative water content	
				Total chlorophyll and chlorophyll a levels increase	
				Peroxidation of lipids was reduced	

Colonization of AMF also affects the concentrations and characteristics of polyamines and organic acids in plants (Sheng et al., 2011). Polyamines by increasing nutrient and water absorption, while organic acids by reducing the electrical conductivity of the soil and increasing the availability of N, P, and K in the soil, helping maintain ionic homeostasis in plant cells (Sheng et al., 2011; Evelin et al., 2019). However, different AMF species have varying abilities to obtain and provide nutrients to salt-stressed plants (Taylor et al., 2015) (**Table 2**).

Adding AMF to plants stressed by salt enhances their growth through allowing them to increase water and nutrient absorption (Pedranzani et al., 2016; Parihar et al., 2020). Researchers have found that AMF in saline environments increases biomass production, shoot and root dry weight, number of branches, plant height, seedling diameter, leaf area and plant yield (Qiu et al., 2007; Zhao et al., 2015; Tao et al., 2012; Parihar et al., 2020). There are several plants whose biomass has increased under saline conditions due to AMF, including *Chrysanthemum morifolium*, *Elaeagnus angustifolia*, *Gossypium hirsutum*, *Medicago sativa*, *Phoenix dactylifera*, *Zelkova serrata*, *Oryza sativa*, *Verbena officinalis*, and *Trigonella foenum-graecum* (Jia et al., 2019; Duc et al., 2021; Evelin et al., 2019). Furthermore, AMF improves plant growth in saline environments by increasing endogenous production of growth regulators like IBA and IAA (Waqas et al., 2012; Abd_Allah et al., 2015). Research conducted by Guo et al. on *Malus domestica Borkh* exposed to salt, *Funneliformis mosseae* inoculation resulted in a positive regulation of genes involved in IAA-responsive proteins in aerial parts (*TRINITY_DN10259_c0_g1*), and roots (*TRINITY_ DN17372_c0_g1*) (Gao et al., 2020). Furthermore, AMF colonization can enhance the plant’s ability to explore water and food through rising the length and conductivity of the roots, and ultimately improve the plant’s adaptation to salinity (Chandrasekaran et al., 2019; Kumar et al., 2010).

Plant roots inoculated with AMF when stressed by salt helps to enhance nitrogen absorption, and studies have shown that up to 25% of plant nitrogen is supplied by AMF hyphae (Evelin et al., 2019; Younesi et al., 2013). The improvement of nitrate uptake by symbiotic plants with AMF could be due to maintaining membrane stability and increasing Nitrate reductase (NR) (Evelin et al., 2019). In plants stressed by salt, coexistence of AMF increases the expression of ammonium transporters (*AMT1.1*, and *AMT1.2*) and nitrate transporters (*NRT1.1*، and *NAR2.2*) (Fileccia et al., 2017; Saia et al., 2015). Meanwhile, under salinity conditions, phosphorus absorption by plants is greatly reduced, while AMF symbiosis is considered as an effective biological method to increase P absorption by plants and stimulate their growth (Romero-Munar et al., 2019; Shokri and Maadi, 2009; Ghorchiani et al., 2018). The findings of a study on *Zelkova serrata* seedlings grown in saline environments indicated that root inoculation with *Funneliformis mosseae* resulted in a noticeable enhance in P levels in root and leaf (Jia et al., 2019). The rise in P uptake in AMF-inoculated plants depends on several factors, including 1) increasing the P availability in soil due to alkaline and acid phosphatases secreted by hyphae, 2) P’s continuous movement into the root, because AMF is able to accumulate large amounts of absorbed P to the root, 3) maintaining the intrinsic concentration of phosphate (Pi) through forming polyphosphates within the hyphae, and 4) AMF is able to absorb P at a lower threshold because of high-affinity phosphate transporter genes expression (*GmosPT*, *GiPT*, and *GvPT*) (Vassilev et al., 2012; Abdel-Fattah and Asrar, 2012; Jia et al., 2019). Furthermore, P’s effective absorption by mycorrhizal plants under salinity stress conditions leads to the following benefits: 1) the selective absorption of ions and thus reducing the adverse effects of salinity, 2) partitioning of toxic ions in vacuoles, 3) reduction of ion leakage, and 4) maintaining cell membrane integrity (Bothe, 2012; Cantrell and Linderman, 2001; Evelin et al., 2019) (**Table 2**).

The researchers observed that the coexistence of AMF rises the absorption of K^+^ in salt-exposed plants, and plant use K^+^ uptake as a coping mechanism to salt stress (Giri et al., 2007; Amanifar et al., 2019). Experiments have demonstrated that inoculating salt-exposed roots with AMF upregulates *SKOR*, resulting in passive secretion of K^+^ in the xylem flow, ultimately increasing K^+^ accumulation in the aerial organs and enhancing K^+^/Na^+^ ratios (Wang et al., 2021; Evelin et al., 2019). A significant increase in K^+^ concentration in the roots and leaves of mycorrhizal seedlings, in addition to preventing disturbances in cellular enzymatic processes and inhibiting protein synthesis, enhances stomatal conductance, enhancing water requirement for transpiration (Garg and Bhandari, 2016; Sheng et al., 2008; Van Zelm et al., 2020). Additionally, the results of several studies indicate AMF helps to overcome salinity-induced deficiencies of Mg2^+^, and Ca2^+^ by an observable rise in the content of these ions in roots and leaves (Navarro et al., 2014; Evelin et al., 2012; Van Zelm et al., 2020) (**Table 2**).

It seems that, in addition to preventing Na^+^ absorption, AMF can reduce salinity’s effects on plants through limiting its transfer to plant aerial parts and also diluting Na^+^ (Janah et al., 2021b; Borde et al., 2011; Talaat and Shawky, 2011). Vesicles in the AMF can store ions such as Na^+^ and Cl^−^ under salt stress, and increase plant adaptation to saline conditions by inhibiting their uptake through roots (Ait-El-Mokhtar et al., 2020; Miransari, 2010). When salt is present, AMF participates in the selective ion absorption such as Mg, N, P, Ca, and K, and reduces the absorption of Na^+^ (Paul and Sinha, 2017; Goussi et al., 2018). Furthermore, mycorrhizal plants can control Na^+^ transport to the upper parts of plant, as well as regulate the internal concentration of Na^+^ (Evelin et al., 2019; Santander et al., 2019). Mycorrhizal plants also reduce the accumulation of Na^+^ in leaves and aerial parts by sequestering Na^+^ in root vacuoles or taking it out of the cytosol (Evelin et al., 2019; Chen et al., 2017). Moreover, AMF assist the host plant in collecting Na^+^ from the xylem and diverting it from the tissues responsible for photosynthesis to the roots (Evelin et al., 2012). Further, there is a possibility that the reduction of ionic toxicity in salinity conditions is due to the dilution effect, since AMF boosts growth and biomass by improving the nutritional status of plants, leading to Na^+^ and Cl^−^ dilution (Talaat and Shawky, 2011; Amanifar et al., 2019).

According to previous studies, plants inoculated with AMF up-regulate aquaporin genes (such as *PIP*), H^+^-ATPase genes (such as *VHA-B*), and Na+/H+ antiporters genes (such as *NHX1* and *SOS1*), and the function of these genes mediates sodium flow and water potential in tissues of plants (Diao et al., 2021; Pedranzani et al., 2016; Evelin et al., 2019; Munns, 2005). Various plant species express these genes differently, in such a way that the expression of *NHX* is regulated by AMF symbiosis in rice under salt conditions, while there is no significant difference for *Robinia pseudoacacia* (Diao et al., 2021). Meanwhile, AMF coexistence increased the expression of *SOS1* in rice shoots and roots of *Robinia pseudoacacia*, but decreased this gene’s expression in root of rice (Chen et al., 2017). In addition, Several studies have shown AMF coexistence in saline environments increases the expression of *PIP1;1*, *PIP2;1*, and *PIP1;3* in *Robinia pseudoacacia*, while it decreases the expression of *PIP1* in tomato roots (Diao et al., 2021; Ouziad et al., 2006).

AMF’s extensive hyphae allow higher hydraulic conductivity, and water absorption capacity even in low water potential and improve soil water availability (Medina and Azcón, 2010; Sheng et al., 2008). In addition, plant root inoculation by AMF in saline environments enhances relative water content (RWC), shoot water content and water use efficiency (WUE) (Al-Khaliel, 2010; Kapoor et al., 2008; Sheng et al., 2008). AMF also rises leaf relative water content (LRWC), and leaf water potential (LWP) in plants grown in saline environments, possibly because it improves water absorption capacity and hydraulic conductivity (Kapoor et al., 2008; Chandrasekaran et al., 2019). The improvement of WUE resulting from root inoculation by AMF resulting in a boost in gas exchange capacity, stomatal conductance and subsequent transpiration in plants stressed by salinity (Elhindi et al., 2017; Sheng et al., 2008; Chandrasekaran et al., 2019).

### 4.2 Enhancement of photosynthetic parameters in salt-stressed plants by AMF

Studies in the past have stated AMF-inoculated plants have shown a higher photosynthetic capacity stressed by salinity (Liang et al., 2021; Abdel Latef and Chaoxing, 2014). AMF can increase photosynthesis of plants suffering from salinity stress by improving transpiration rate, stomatal conductance, water status of leaves, strengthen of photosynthesis machinery, pigment content, photochemistry and non-photochemistry of PSII, carbon uptake and transfer to mycorrhizae (Zai et al., 2021; Cho et al., 2006; Ait-El-Mokhtar et al., 2020). The coexistence of AMF in salty environments affects age-related changes (ARCs) in metabolome of leaves and partially prevents leaf aging and leads to the better metabolites accumulation (Shtark et al., 2019). In addition, AMF treatment protects leaf cells of saline-grown plants by preventing cell wall detachment, reducing plasma membrane damage, inhibiting chloroplasts vanish, stopping thylakoid destruction, and maintaining chloroplast structure (Liang et al., 2021; Evelin et al., 2013). Less damage to chloroplast structure caused by AMF inoculation might be as a result of a higher osmolyte concentration (sugars, betaine, proline, glycine) and polyamines, and larger and more plastoglobules (higher concentration of alpha-tocopherol) in plants (Evelin et al., 2013; Talaat and Shawky, 2014). Osmolyte accumulation in mycorrhizal plants protects CO_2_ fixing enzymes [rubisco activase (Rca), and PSII pigment protein complexes ribulose-1,5-bisphosphate (RuBisCO)] (Talaat and Shawky, 2014). Plastoglobules, as tocopherol synthesis sites, also can play a protective role in membrane of thylakoids and proteins (Austin et al., 2006). However, tocopherol probably protects PSII by preventing photooxidation of membrane lipids (Liang et al., 2021). AMF leads to the upregulation of the expression of chloroplast-related genes (*RppsbA*, and *RppsbD*), which gives the plant a higher PSII efficiency and then boosts the photosynthetic capacity under salinity stress (Chen et al., 2017). Furthermore, AMF treatment improves photosynthetic capacity by increasing N absorption, because N is considered to be a major component of Rubisco enzymes (Ahanger et al., 2014) (**Figure 4**; **Table 2**).

AMF inoculation of plant roots reduces the adverse effects of salinity on chlorophyll content and photosynthetic pigments by removing toxic Na^+^ and inhibiting its transfer to aerial organs (Abeer et al., 2014; Chandrasekaran et al., 2019; Khalil et al., 2011). AMF can also promote chlorophyll production and accelerate photosynthetic activity by improving the chlorophyll synthetase enzyme activity (Wright et al., 1998; Liang et al., 2021). The study conducted on *Echinacea angustifolia* has shown that the inoculation of plant roots by *Rhizophagus irregularis* under salinity stress increased the chlorophyll content of leaves 2 to 3 times more than the treatments without AMF (Liang et al., 2021). Liang et al. also demonstrated that AMF treatment gave a much greater increase in Chl a content compared to Chl b and carotenoids, such that the AMF-induced improvement in Chl a was 25%, however, it was less than 20% for Chl b (Liang et al., 2021). The results of the studies conducted on *Sesbania sesban*, *Solanum lycopersicum*, *Zelkova serrata*, *Panicum turgidum*, *Arundo donax*, and *Ocimum basilicum* grown in saline environments also indicated that the inoculation of roots by AMF species increases the photosynthetic pigments and chlorophyll content (Elhindi et al., 2017; Liang et al., 2021; Pollastri et al., 2018; Wang et al., 2019b). Additionally, research has shown that the mediating AMF’s effect in saline environments on leaf surface, photosynthesis rate, total chlorophyll, Chl a and Chl b in C3 plants is more than C4 plants (Chandrasekaran et al., 2019). Since there is a reciprocal relationship between Soil-Plant Analysis Development (SPAD), leaf nitrogen content, and total chlorophyll, research has shown that colonization of AMF in saline conditions increases SPAD (Campanelli et al., 2013; Porcel et al., 2015; Wu et al., 2007). Moreover, AMF application enhances the absorption of Mg2^+^ by plants in saline environments, and since the Mg2^+^ ion is the chlorophyll molecule’s central ion, it boosts the content of chlorophyll in the inoculated plants (Rao and Chaitanya, 2016; Jia et al., 2019). Mg2^+^ is also necessary for the appropriate function of several enzymes such as glutathione synthase, protein kinases, ATPases, carboxylases, phosphatases, and RNA polymerases (Shaul, 2002).

It is well known that fixation of photosynthetic CO_2_ is essential for rapid plant growth process, and is extremely sensitive to changes in the environment, including salt stress (Liang and Shi, 2021). Research has indicated that a higher content of photosynthetic pigments is the basis of better photosynthetic gas exchange, and since plant-AMF symbiosis increases chlorophyll content and photosynthetic pigments, thus also improving gas exchange and carbon fixation (Takai et al., 2010; Elhindi et al., 2017). The response of mycorrhizal plants under salinity stress can be complicated in relation to gas exchange, so that the results of studies have shown that although CO_2_ exchange increases, however, plants face a decrease in intercellular CO_2_ concentration by increasing photosynthesis, which is due to the effective use of CO_2_ with the coexistence of AMF (Sheng et al., 2008; Chandrasekaran et al., 2019). Further, the researchers’ results have shown that the coexistence of plants with AMF in saline environments reduces stomatal conductance and transpiration rate (Liang and Shi, 2021). Research also has shown that although C3 plants show higher photosynthesis rate and transpiration rate than C4 plants, however, C4 plants have a higher amplitude in stomatal conductance, WUE, and RWC (Pinto et al., 2014; Chandrasekaran et al., 2019). Additionally, there is a significant relationship among stomatal conductance and photosynthetic capacity (A_max_) in C4 plants inoculated with AMF in salty conditions, so that increasing stomatal conductance indicates the enhancement of A_max_ (Chandrasekaran et al., 2019).

Using AMF in saline environments can improve the ability of plants to dissipate excessive energy through increasing the regulation of energy splitting among photochemical and non-photochemical events and protect the photosynthetic apparatus from excessive light (Sheng et al., 2008; Jia et al., 2019). Increasing photochemical efficiency in plants improves CO_2_ fixation, photosynthetic activities, Rubisco activities, water status of plants, and stomatal conductance (Gao et al., 2020; Porcel et al., 2015; Navarro et al., 2014). Additionally, AMF balances the absorption and use of light energy to obtain photoprotection and reduces salt stress damage to PSII by decreasing the values ​​of ФPSII (actual PSII efficiency), and qP (photochemical quenching) (Zribi et al., 2009; Chen et al., 2017; Hanachi et al., 2014; Ait-El-Mokhtar et al., 2019). When the salinity is high, AMF inoculation’s positive effects on the level of PSII photoinhibition decreases, which can be due to the host plant ionic imbalance and ultimately disrupting the normal cellular function (Jia et al., 2019). Moreover, several studies have found increasing in non-photochemical quenching (NPQ) in leaves of AMF-inoculated plants in salt-stressed conditions, which is an energy dissipation mechanism that provides protection for the photosynthetic apparatus from excess light under salinity (Jia et al., 2019; Zribi et al., 2009). However, AMF’s effects in saline environments on NPQ was different, so that it increased in *Zea mays* leaves, while it decreased in *Cucumis melo* leaves and did not show any change in *Robinia pseudoacacia* leaves (Jia et al., 2019). AMF also up-regulates the synthesizing genes expression of abscisic acid 8′-hydroxylase, capsanthin/capsorubicin synthase and NAD(P)H-ubiquinone oxidoreductase to enhance the photoprotection mechanisms of chloroplasts (Begum et al., 2019) (**Table 2**).

### 4.3 Amplification of defense mechanisms in salt-stressed plants by AMF

AMF reduces ROS, oxidative damage and cell leakage in salt-exposed plants by enhancing the enzymatic, and non-enzymatic antioxidant defense system, Phytohormone synthesis, solute accumulation, stimulation of synthesis of osmolytes, and protection of membrane lipids (Parihar et al., 2020; Bistgani et al., 2019; Begum et al., 2019). In addition, the inoculation of plants grown in saline environments by AMF reduces oxidative stress markers (MDA, and H_2_O_2_), and lipid peroxidation (Ait-El-Mokhtar et al., 2020; Gao et al., 2020). Some studies have shown that the content of H_2_O_2_ and MDA with AMF inoculation is variable in different plant organs, such that the leaves have a lower content of H_2_O_2_ and MDA while the roots have a higher level of them, which is due to more Na^+^ accumulation in the roots (Chen et al., 2017, 2020; Sewelam et al., 2016) (**Table 2**).

**Figure 5:**
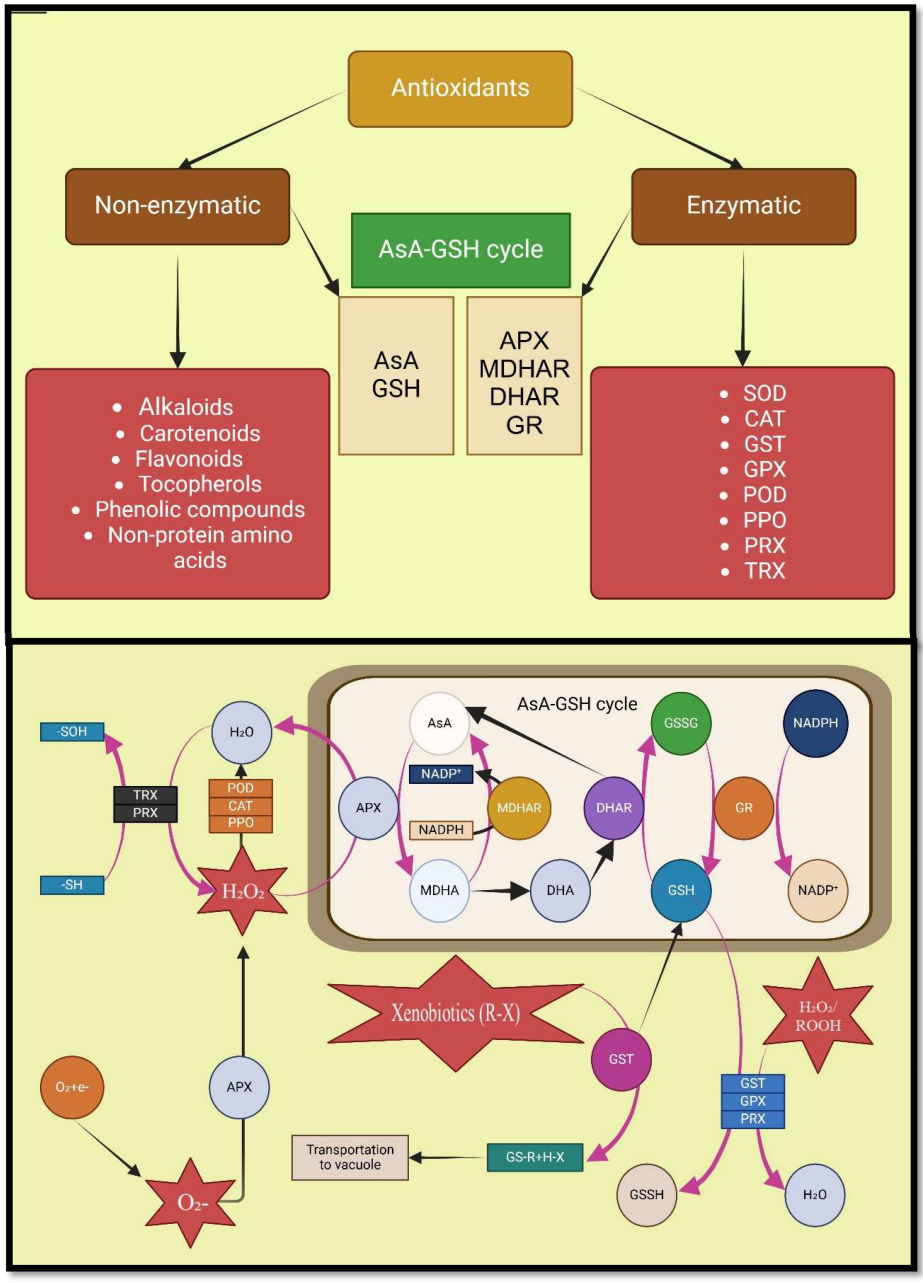
Mechanisms involved in action of various antioxidants. Nomenclature is as proposed by Hasanuzzaman et al. (2021).

During saline conditions, symbiosis with AMF reduces the threshold concentration of ROS required to cause oxidative degradation by increasing antioxidants activities such as APX, CAT, SOD, POD, GSH, monodehydroascorbate reductase (MDHAR), dehydroascorbate reductase (DHAR), POX, glutathione reductase (GR), and AsA (Bose et al., 2014; Shahvali et al., 2020; Ait-El-Mokhtar et al., 2020; Apel and Hirt, 2004; Samaddar et al., 2019). In their study on *Robinia pseudoacacia* stressed by salt, Chen et al. showed that root inoculation by *Rhizophagus irregularis* caused antioxidant enzyme gene expression (*RpMn-SOD*, *RpCu/Zn-SOD*, *RpAPX2*, *RpAPX1*, *RpGR*), which resulted in the reduction of H_2_O_2_, MDA, and REL in the leaves (Chen et al., 2020). Coexistence with AMF through increasing the secondary metabolites accumulation in tissues of plants causes morphophysiological and hormonal changes in host plants, including removal of ROS, and production of antioxidant (Pan et al., 2020; de Lazzari Almeida et al., 2018; Evelin et al., 2013). Furthermore, according to studies, AMF not only increases secondary metabolite production and accumulation in medicinal plants under salt stress, but also improves their medicinal value (Zeng et al., 2013; Amanifar et al., 2019) (**Figure 5**; **Table 2**).

Phenols are one of the bioactive compounds that increases in medicinal plants organs by AMF inoculating (Kapoor et al., 2017). Among the polyphenols that rise under the influence of AMF are quercetin-3-arabinoside, luteolin, 4-O-caffeoylquinic acid, 4,5-dicaffeoylquinic acid, and protocatechuic (Aseel et al., 2019). However, in a salt-salt stressed mycorrhizal plant, conflicting observations have been published about phenolic compounds accumulation (Duc et al., 2021). Although research conducted on *Echinacea angustifolia* showed that mycorrhization increased flavonoids’ total content, the research performed on lettuce leaves identified that the content of phenol decreased significantly (Liang et al., 2021; Santander et al., 2019). Nevertheless, according to Duke et al., these differences are due to the fact that the majority of prior research investigated polyphenol profiles only at a harvest, while polyphenol content varies with plant age (Duc et al., 2021). Additionally, researchers have found that stress intensity affects both increasing and decreasing trends in phenolic compounds in plants colonized by AMF, and the best performance of AMF is in moderate salt stress (Duc et al., 2021; Amanifar and Toghranegar, 2020). Moreover, changes in the metabolism of carbohydrates and primary metabolites, which can be caused by the improvement of nutrients, water absorption and photosynthesis in colonized plants, enhance Phenolic compounds (Arzani and Ashraf, 2016; Pedone-Bonfim et al., 2018).

The synthesis mechanisms of proline, as a proteinogenic amino acid, can be improved by AMF colonization and enhance tolerance of plants to salt (Abd_Allah et al., 2015; Ahanger et al., 2013). Proline reduces the risk of salt stress by preventing free radical damage, maintaining osmotic balance, protein degradation induced by stress, redox enzyme stabilization, protecting membrane integrity, improving cell water retention, and increasing K concentration in the cell (Duc et al., 2021; Lin et al., 2016; Reddy et al., 2015; Ait-El-Mokhtar et al., 2020; Parihar et al., 2020). Plants’ increased proline content can be attributed to factors, such as 1) inactivation of proline dehydrogenase (catalyzes the breakdown of proline), 2) a greater glutamate dehydrogenase enzyme activity (involved in glutamate synthesis, a precursor of proline), 3) a higher level of Pyrroline-5-carboxylate synthase (P5CS), and 4) increasing in P5CS gene expression (Abo-Doma et al., 2011; Garg and Baher, 2013). Nevertheless, studies on the effects of AMF on the concentration of proline in plants under salinity have been contradictory, although several researches have informed higher content of proline in mycorrhizal plants, while others have shown lower proline in plants (Tuo et al., 2015; Elhindi et al., 2017; Shekoofeh et al., 2012; Yooyongwech et al., 2016). Since the proline molecule is regarded as a stress marker, the reduction of proline synthesis in mycorrhizal plants possibly as a result of AMF-mediated stress reduction (Duc et al., 2021; Evelin et al., 2019).

Organic acids are essential osmolytes in the vacuoles of plants, and AMF colonization plays an important involvement in regulating their concentrations and metabolism, increasing tolerance to salinity in plants (Guo et al., 2010; Evelin et al., 2019). A number of organic acids are increased in mycorrhizal plants stressed by salt, including oxalic, malic, fumaric, citric, acetic acids (Sheng et al., 2011). Moreover, plants with AMF symbiosis accumulate more soluble carbohydrates that reduce the harm resulting from excessive salt exposure, through stabilizing the structure and activities of protein complexes, membrane integrity, maintaining the activity of mature leaves, and balancing energy transfer (Gao et al., 2020; Dodd and Pérez-Alfocea, 2012; Diao et al., 2021). Trehalose (α-D-glucopyranosyl-1,1-α-D-glucopyranoside) as a non-reducing disaccharide controls carbohydrate metabolism, and AMF symbiosis can increase this osmolyte accumulation in plants in saline environments (Lunn et al., 2014; Chang et al., 2014). The higher concentration of trehalose in mycorrhizal plants can be assigned to the increased Trehalose-6-phosphate phosphatase (TPP), and Trehalose-6-phosphate synthase (TPS) activities (enzymes responsible for trehalose biosynthesis) by AMF and the decreased TRE activity (trehalose-degrading enzymes, including Trehalase, and Trehalose phosphorylase) (Garg and Bhandari, 2016).

On the other hand, studies have shown that in roots inoculated with AMF, glycyrrhizin biosynthesis is rised in salinity-stressed conditions, that reduces oxidative stress (Orujei et al., 2013; Amanifar et al., 2019). Further, the observed increase in the concentration of glycyrrhizin in mycorrhizal plants might be due to the rise in enzyme molecule number, as well as the boost in the production of terpenoid biosynthesis precursors (Amanifar et al., 2019). A rise in P uptake by mycorrhizal plants results in a rise in the biosynthesis of terpenoids through enhancing the production of pyrophosphate compounds such as dimethylallyl diphosphate (DMAPP), and isopentenyl pyrophosphate (IPP), which are key precursor molecules for the biosynthesis of terpenoids (Xie et al., 2018). In addition, P contribution in the formation of other precursors of terpenoids through MVA (mevalonic acid) [NADPH (Nicotinamide adenine dinucleotide phosphate), ATP (Adenosine triphosphate), and acetyl-CoA (Acetyl coenzyme A)], and MEP (phosphoglyceraldehyde and pyruvate) pathways is crucial (Kapoor et al., 2017). As the major component of glycyrrhizin biosynthesis, farnesyl pyrophosphate (FPP) is synthesized with IPP and DMAPP condensation sequentially (Kapoor et al., 2017) (**Table 2**).

## 5 Conclusions

The review showed that salinity stress can be reduced in plants by using AMF. AMF strengthens plants’ resistance to salinity by enhancing the absorption of nutrients, and water, selective absorption of elements, the photosynthetic apparatus and the antioxidant defense mechanisms. However, the present review indicates that more studies are needed in various fields, including 1) the role of AMF in reducing salinity stress in field experiments, 2) the contribution of different AMF species in improving plant biochemical and molecular structures under salt stress, 3) interaction of different AMF species in saline environments, 4) the effect of AMF on soil physical and chemical structures, as well as plant root architecture, and 5) AMF’s role in simultaneously reducing salinity stress and other abiotic, and biotic stresses.
